# Some Observations on the Mental State of the Blind and Deaf and Dumb; Suggested by the Case of Jane Sullivan, Both Blind, Deaf, Dumb and Uneducated

**Published:** 1844-01

**Authors:** 


Art. II.—*Some Observations on the Mental State of the Blind and Deaf and Dumb; suggested by the case of Jane Sullivan, both blind, deaf, dumb, and uneducated.* By R. Fowler, m.d. f.r.s.—*Salisbury*, 1843. 12mo, pp. 64.

This little pamphlet is well worth the perusal of those who feel an interest in the remarkable class of cases of which it treats; and as the public mind has been lately much directed towards the American girl, Laura Bridgeman, whose remarkable progress has been due to the untiring exertions and intelligent guidance of Dr. Howe, the notices collected by Dr. Fowler, of several cases presenting the same deficiency, but in which no attempt at education had been made, are additionally valuable, for the sake of comparison. He considers that, from observations made upon such cases, important inferences may be drawn, in regard to the *muscular sense*, to which he attributes that preparation or adjustment of the organs of special sense for the ready reception of impressions, which takes place when the *attention* is directed to the sensations they convey.

